# Protective role of *Phyllantus niruri* extract in doxorubicin-induced myocardial toxicity in rats

**DOI:** 10.4103/0253-7613.75663

**Published:** 2011-02

**Authors:** A.H.M. Thippeswamy, Akshay Shirodkar, B.C. Koti, A. Jaffar Sadiq, D.M. Praveen, A.H.M. Viswanatha Swamy, Mahesh Patil

**Affiliations:** Department of Pharmacology, K.L.E. University’s College of Pharmacy, Hubli - 580 031, Karnataka, India; 1 Department of Pharmaceutical Chemistry, K.L.E. University’s College of Pharmacy, Hubli - 580 031, Karnataka, India

**Keywords:** *Phyllanthus niruri*, antioxidant, cardiotoxicity, doxorubicin

## Abstract

**Objectives:**

To investigate the effect of the aqueous extract of *Phyllanthus niruri* (Aq.E.PN) against doxorubicin (Dox)-induced myocardial toxicity in rats.

**Materials and Methods:**

Cardiotoxicity was produced by Dox administration (15 mg/kg for 2 weeks). Aq.E PN (200 mg/kg, orally) was administered as pretreatment for 2 weeks alternated with Dox for the next 2 weeks. The general observations, mortality, histopathology, biomarker enzymes like lactate dehydrogenase (LDH), creatinine phosphokinase (CPK) and alkaline phosphatase, diagnostic enzyme markers like aspartate aminotransferase (AST) and alanine aminotransferase (ALT), and antioxidants such as glutathione (GSH), superoxide dismutase (SOD), catalase (CAT) and malondialdehyde (MDA) were monitored after 3 weeks of the last dose.

**Results:**

Pretreatment with the Aq.E.PN significantly (*P* < 0.01) protected the myocardium from the toxic effects of Dox by reducing the elevated level of biomarker and diagnostic enzymes like LDH, CPK, AST and ALT to the normal levels. Aq.E PN increased the GSH, SOD and CAT levels and decreased the MDA levels in cardiac tissue. Administration of Dox caused cardiomyopathy associated with an antioxidant deficiency.

**Conclusion:**

These results suggest a cardioprotective effect of *P. niruri* due to its antioxidant properties.

## Introduction

Doxorubicin (Dox) or Adriamycin is a clinically well-established anticancer drug that is widely used for the treatment of various neoplastic diseases, including breast cancer, acute leukemias and Hodgkin and non-Hodgkin lymphoma, etc. However, the clinical use is restricted by an unusual and often irreversible dose-dependent cardiomyopathy.[1] The Dox-induced cardiotoxicity has been shown to be mediated through different mechanisms, including membrane lipid peroxidation,[2] free radical formation,[3] mitochondrial damage[4] and decreased activity of Na^+^–K^+^adenosine triphosphate.[5]

The species *Phyllanthus niruri* (Linn.), also known as *Phyllanthus amarus*, is a traditional herbaceous plant distributed all over India. It has been reported to have hypotensive,[6] antiulcerogenic,[7] antitumor,[8] antioxidant and hepatoprotective,[9] wound healing[10] and antiamnesic[11] properties. The hepatoprotective and antioxidant activity has been attributed to the presence of phytochemicals like phyllanthin and hypophyllanthin and flavonoids like niruriflavone, gallic acid and ellagic acids.[12] Considering the myriads of phytochemicals in *P. niruri*, the aim of this study was to evaluate the antioxidant and cardioprotective properties of the aqueous extract of *P. niruri* on Dox-induced cardiotoxicity.

## Materials and Methods

### 

#### Plant material

The whole plant of *P. niruri* Linn. was collected from Hubli and its surrounding areas, Karnataka, India. The plant was identified and authenticated by the Botany Department of H.S.K. Science Institute, Hubli. The plant material was dried at room temperature and subjected to coarse powder of desired particle size.

#### Preparation of the plant extract

Weighed quantity of powdered plant material (25 g) was soaked in boiling water (250 ml) for 15 min, allowed to cool and filtered using Whatman filter paper. The obtained residues were further extracted twice and then concentrated using a rotary evaporator. The concentrated extract was then taken in a china dish and evaporated on a thermostat-controlled water bath till it formed a thick paste.

#### Animals

Healthy albino Wistar rats of either sex weighing between 150 and 200 g of 10–12 weeks of age were used. Animals were housed individually in polypropylene cages, maintained under standard conditions (12:12 L:D cycle; 25 ± 3°C and 35–60% humidity), fed with standard rat pellet diet, (Hindustan Lever Ltd, Mumbai, India.) and water *ad libitum*. The study was approved by the institutional animal ethics committee (KLESCOPH / IAEC. Clear / 2007-08).

#### Materials

Dox was procured from Dr. R. B. Patil Cancer Research Hospital, Hubli, India. Other chemicals used were of analytical grade and were procured locally. Analyzing kits were obtained from ERBA Diagnostics, Daman, India.

#### Acute toxicity study

The acute oral toxicity study was carried out as per the revised guidelines by Organization for Economic Co-Operation and Development (OECD) and Committee for the Purpose of Control and Supervision of Experiments on Animals (CPCSEA). Acute toxicity studies were performed on albino mice weighing between 20 and 30 g using the up and down method employed prior to evaluating the cardioprotective activity of *P. niruri*.

#### Experimental design

After 1 week of acclimatization, the animals were randomly divided into four groups of six animals each. Group 1 served as normal control and received normal saline 5 ml/kg body weight (i.p.). Group 2 was treated with Dox (2.5 mg/kg body weight i.p.) in six equal injections alternatively for 2 weeks to make a total cumulative dose of 15 mg/kg body weight. Group 3 received Aq.E. PN (200 mg/kg body weight p.o.) for 2 weeks and then alternatively with vehicle for the next 2 weeks. Group 4 was pretreated with Aq.E. PN 200 mg/kg body weight p.o. for 2 weeks followed by Dox administration as in group 2.

#### Enzyme assays

Thirty-six hours after the last treatment, orbital blood samples were obtained under light ether anesthesia using heparinized microcapillaries for the estimation of biomarkers lactate dehydrogenase (LDH),[13] creatinine phosphokinase (CPK)[14] and alkaline phosphatase (ALP).[15] Both control and treated animals were observed for 3 weeks after the last injection for general appearance, behavior and mortality. At the end of 3 weeks posttreatment period, animals were sacrificed under ether anesthesia and the heart tissue was quickly dissected, washed in ice cold saline, dried on filter paper and weighed immediately. A portion of each heart was taken from all the groups and a 30% w/v homogenate was prepared in 0.9% buffered KCl (pH 7.4) for the estimation of glutathione (GSH),[16] malondialdehyde (MDA),[17] superoxide dismutase (SOD),[18] catalase (CAT),[19] aspartate aminotransferase (AST) and alanine aminotransferase (ALT).[20] The remaining portion of the heart tissue was used for histopathological studies.

#### Statistical analysis

The results were expressed as the mean ± SEM and analyzed using one-way ANOVA followed by Dunnett’s multiple comparison tests. Data were computed for statistical analysis using the Graph Pad Prism Software.

## Results

Chronic administration of Dox induced cardiac toxicity and effect of Aq.E PN was established by measuring cardiac biomarker enzymes, endogenous antioxidants and heart tissue histopathology. Acute toxicity studies observed that a maximum dose of 2000 mg/kg b.w. was safe in animals. However, few changes in the behavioral response, like alertness, touch and restlessness, were noted. Therefore, 1/10^th^of the maximum tolerated dose, 200 mg/kg b.w., was chosen for further studies.

### 

#### Heart weight, body weight and ratio of heart weight to body weight

The heart weight, body weight and ratio of heart to body weight was significantly (*P* < 0.01) increased compared with normal rats in group 2, and significantly (*P* < 0.01) decreased in group 4 as compared with the Dox-treated group [Table 1].

**Table 1:** Efficacy of Aq.E. PN on heart weight, body weight, ratio of heart weight to body weight and cardiac markers in doxorubicin-induced cardiotoxicity in rats (n = 6)

*Treatment*	*Body weight (g)*	*Heart weight (g)*	*Heart weight/body weight ratio (^×^10-3)*	*CPK (IU/L)*	*LDH (IU/L)*
Group 1 (normal saline 5 ml/kg)	194.2 ± 3.96	0.61 ± 0.01	3.14	154.7 ± 4.599	239.0 ± 1.818
Group 2 (Dox)	163.3 ± 2.47^a^	0.72 ± 0.59^a^	4.40	328.9 ± 1.605^a^	416.1 ± 2.316^a^
Group 3 (Aq.E. PN)	197.5 ± 2.14	0.59 ± 0.02	2.98	154.2 ± 1.571	256.5 ± 2.808
Group 4 (Aq.E. PN + Dox)	184.2 ± 3.00^b^	0.65 ± 0.01^b^	3.52	230.2 ± 1.356^b^	325.8 ± 3.617^b^

The results are expressed as mean ± SEM. Significance was calculated by one-way ANOVA with Dunnett’s “t” test. Dox: doxorubicin, 2.5 mg/kg, i. p. in six equal doses on alternate days for 2 weeks. Aq.E. PN: Aqueous extract of *Phyllanthus niruri* 200 mg/kg, p.o. for 2 weeks followed by Dox

a *P* < 0.01 when compared with normal.

b *P* < 0.01 when compared with doxorubicin; CPK, creatinine phosphokinase; LDH, lactate dehydrogenase

#### Cardiac markers

Animals treated with Dox produced a significant (*P* < 0.01) increase in the level of LDH and CPK as compared with the rats in group 1 [Table 1]. Pretreatment with the Aq.E. PN extract decreased the LDH and CPK level as compared with group 2.

#### Serum enzyme biomarkers

Animals treated with Dox produced a significant (*P* < 0.01) increase in the level of AST, ALT and ALP as compared with group 1 [Table 2]. Pretreatment with the Aq.E. PN extract inhibited the AST, ALT and ALP levels as compared with group 2.

**Table 2:** Effect of Aq.E. PN on AST, ALT and ALP level in doxorubicininduced cardiotoxicity in rats (n = 6)

*Treatment*	*AST (IU/L)*	*ALT (IU/L)*	*ALP (IU/L)*
Group 1 (normal saline 5 ml/kg)	63.92 ± 0.6749	29.08 ± 1.125	118.9 ± 0.6484
Group 2 (Dox)	188.1 ± 2.796^a^	57.57 ± 1.972^a^	242.7 ± 1.424^a^
Group 3 (Aq.E. PN)	67.83 ± 1.747	30.27 ± 0.7399	116.7 ± 1.144
Group 4 (Aq.E. PN + Dox)	103.4 ± 1.499^b^	45.86 ± 1.505^b^	199.3 ± 1.335^b^

The results are expressed as mean ± SEM. Significance was calculated by one-way ANOVA with Dunnett’s “t” test. Dox: Doxorubicin, 2.5 mg/kg, i.p. in six equal doses on alternate days for 2 weeks. Aq.E. PN: aqueous extract of *Phyllanthus niruri*, 200 mg/kg, p.o. for 2 weeks followed by Dox.

a *P* < 0.01 when compared with normal.

b *P* < 0.01 when compared with Dox; AST, aspartate aminotransferase; ALT, alanine aminotransferase; ALP, alkaline phosphatase

#### Antioxidant status

The MDA level was increased whereas the GSH, SOD and CAT levels were significantly (*P* < 00.01) decreased in the Dox-treated group as compared with normal animals [Table 3]. Group 4 produced a significant decrease in MDA and increased antioxidant enzymes.

**Table 3:** Effect of Aq.E. PN on malondialdehyde, glutathione, catalase and superoxide dismutase in doxorubicin-induced cardiotoxicity in rats (n = 6)

*Treatment*	*Malondialdehyde (nmol of MDA/min) g of wet gland*	*Glutathione (nmol/min/g of heart tissue)*	*Catalase activity (µmole H2O2 decomposed/min/mg protein)*	*Superoxide dismutase activity (units/min/mg protein)*
Group 1 (normal saline 5 ml/kg)	18.33 ± 0.6921	2.74 ± 0.1536	59.04 ± 1.596	37.01 ± 0.6598
Group 2 (Dox)	49.64 ± 2.021^a^	1.527 ± 0.1632^a^	40.75 ± 1.404^a^	22.78 ± 0.7568^a^
Group 3 (Aq.E. PN)	19.41 ± 1.785	2.835 ± 0.1301	60.84 ±1.518	39.07 ± 0.8142
Group 4 (Aq.E. PN + Dox)	25.86 ± 0.7463^b^	1.715 ± 0.0935^b^	50.45±0.9790^b^	31.26 ± 0.5869^b^

The results are expressed as mean ± SEM. Significance was calculated by one-way ANOVA with Dunnett’s “t” test. Dox: Doxorubicin, 2.5 mg/kg, i.p. in six equal doses on alternate days for 2 weeks. Aq.E. PN: Aqueous extract of *Phyllanthus niruri*, 200 mg/kg, p.o. for 2 weeks followed by Dox

a *P* < 0.01 when compared with normal.

b *P* < 0.01 when compared with Dox

#### Histopathological observation

**Figure 1:**
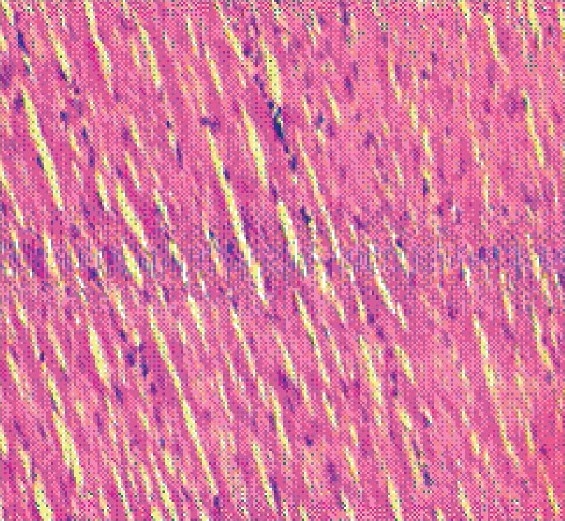
Group 1- showing normal myocardial fibres and architecture (x10)

**Figure 2:**
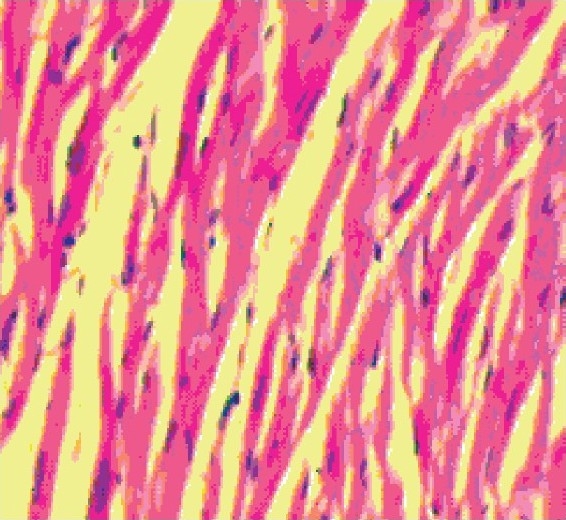
Group 2- Doxorubicin treated group showing loss of myocardial fibres and vacuolated cells (×10)

**Figure 3:**
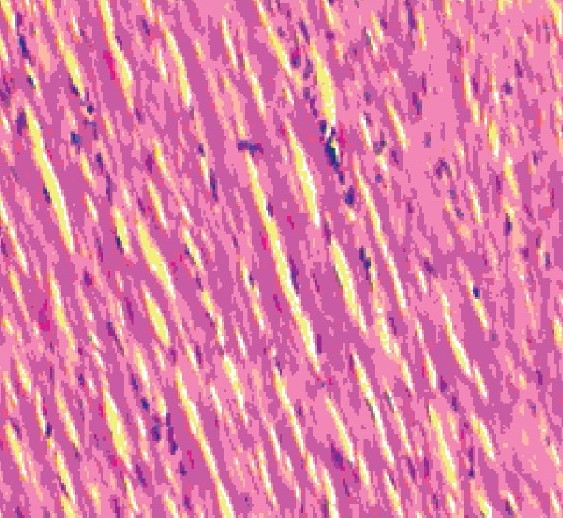
Group 3- Aq.E.PN treated group showing no vacuolated cells and myofibres loss and normal architecture (×10)

**Figure 4:**
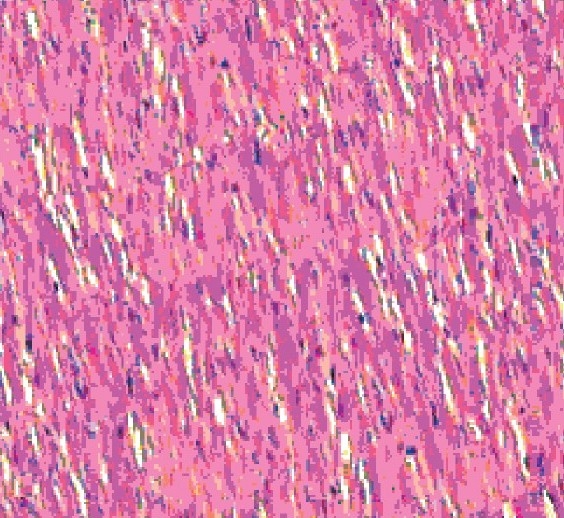
Group 4-Aq.E.PN + doxorubicin treated group showing scanty myocardial fibres loss and vacuolated cells (×10)

The histology of the heart tissue from the control and the Aq.E. PN treated animals showed normal morphological appearances [Figures 1 and 3], whereas in group 2 loss of myofibrils, vacuolization of the cytoplasm, enlarged swollen mitochondria, patchy necrosis and inflammatory cells were observed [Figure 2]. The histology of heart tissues from group 4 showed less loss of myofibrils and vacuolization of the cytoplasm [Figure 4].

## Discussion

The existing experimental evidence suggests that Dox oxidative stress is due to the generation of free radicals in the heart tissue.[21] Heart tissue is especially susceptible to free radical injury because of low levels of free radical detoxifying enzymes like SOD, CAT and GSH. Further, Dox also has a high affinity for the phospholipid component of the mitochondrial membrane in cardiac myocytes, leading to accumulation of Dox in the heart tissue.[22] Cellular GSH depletion is closely related to the lipid peroxidation and disturbance of Ca^2+^ influx induced by toxic agents. Oral administration of the Aq.E. PN extract along with Dox maintained the concentration of GSH at near-normal levels, which prevents cell disruption probably by decreasing the Ca^2+^influx.

The present study has shown that Dox induces lipid peroxidation and decreases the levels of protective enzymes in the heart tissues. Pretreatment with Aq.E. PN significantly reduced the lipid peroxidation and increased the levels of SOD, CAT and GSH. These results indicate the protective effect of Aq.E. PN on Dox-induced cardiotoxicity by scavenging of free radicals.

The antioxidant effects of plant species in the genus Phyllanthus have been reported. For example, *P. urinaria* and *P. emblica* reduced the oxidative damage in Dox-induced cardiotoxicity[23] and ischemic-reperfusion injury,[24] respectively. This may be due to the presence of antioxidants such as flavonoids and other phenolic compounds. The phyllanthin, hypophyllanthin and flavonoids, like niruriflavone, gallic acid and ellagic acids, triterpenoids and phenolic compounds present in the *P. niruri* may have different functional properties, such as scavenging of reactive oxygen species, inhibition of generation of free radicals and chain-breaking activity. This may act as hydrogen-donating radical scavenger by scavenging lipid alkoxyl and peroxyl radical and protect the myocardium from Dox-induced injury.

A deficiency of oxygen supply or glucose may damage the myocardial cells and the cell membrane becomes permeable or ruptures, resulting in leakage of enzymes. We observed an increase in the activities of LDH, CPK, AST and ALT in Dox-treated rats. Pretreatment with Aq.E. PN decreased the enzyme activities in serum and increased the same in the heart. Similar results have been observed by Koti *et al*.[25] This could be due to the protective or membrane-stabilizing effect of Aq.E. PN on the myocardium, reducing the cardiac damage and, thereby, restricting the leakage of these enzymes. In addition, Dox-induced cardiotoxicity is also characterized by decreased body weight and increase in the heart weight. The results of the present study confirmed the earlier findings that Dox administration caused a decrease in the body weight and increase in the heart weight. The histopathological report suggests that the *P. niruri* pretreated group attenuates the Dox-induced loss of myofibrils, vacuolization of the cytoplasm and swelling of mitochondria. Phyllanthin, hypophyllanthin, triterpenoids, niruriflavone and phenolic compounds present in *P. niruri* may be responsible for reducing the oxidative damage.

## Conclusion

In conclusion, the present results suggest that *P. niruri* prevents the Dox-induced myocardial toxicity by boosting the endogenous antioxidant activity. Further studies are needed to elucidate the exact mechanism of action of *P. niruri* and its clinical application.
